# Membrane Design Principles for Ion-Selective Electrodialysis: An Analysis for Li/Mg Separation

**DOI:** 10.1021/acs.est.3c08956

**Published:** 2024-02-07

**Authors:** Ruoyu Wang, Shihong Lin

**Affiliations:** † Department of Civil and Environmental Engineering, Vanderbilt University, Nashville, Tennessee 37235-1831, United States; ‡ Department of Chemical and Biomolecular Engineering, Vanderbilt University, Nashville, Tennessee 37235-1831, United States

**Keywords:** Li/Mg selectivity, selective ion exchange membranes, selective electrodialysis, nanofiltration

## Abstract

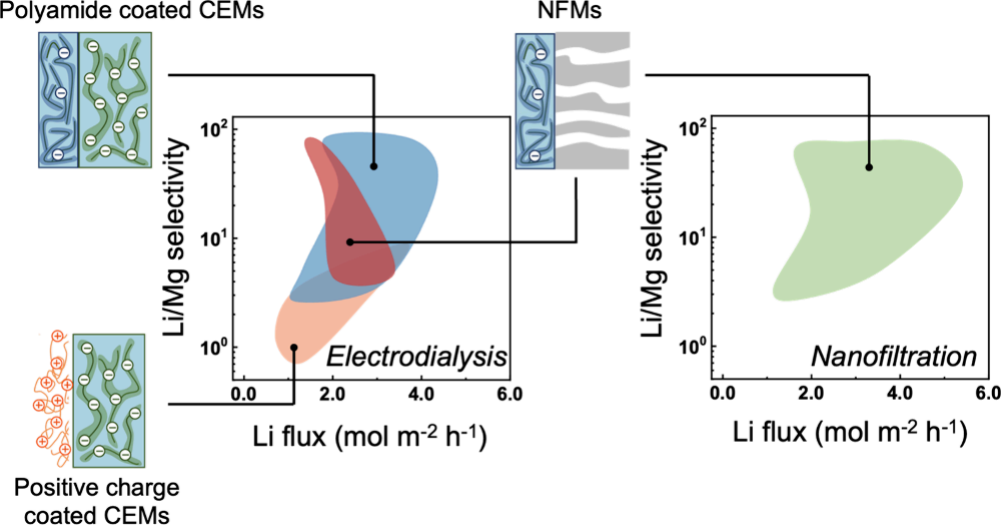

Selective electrodialysis (ED) is a promising membrane-based process to separate Li^+^ from Mg^2+^, which is the most critical step for Li extraction from brine lakes. This study theoretically compares the ED-based Li/Mg separation performance of different monovalent selective cation exchange membranes (CEMs) and nanofiltration (NF) membranes at the coupon scale using a unified mass transport model, i.e., a solution-friction model. We demonstrated that monovalent selective CEMs with a dense surface thin film like a polyamide film are more effective in enhancing the Li/Mg separation performance than those with a loose but highly charged thin film. Polyamide film-coated CEMs when used in ED have a performance similar to that of polyamide-based NF membranes when used in NF. NF membranes, when expected to replace monovalent selective CEMs in ED for Li/Mg separation, will require a thin support layer with low tortuosity and high porosity to reduce the internal concentration polarization. The coupon-scale performance analysis and comparison provide new insights into the design of composite membranes used for ED-based selective ion–ion separation.

## Abstract 2

Selective lithium magnesium separation via electrodialysis requires the design of cation exchange membranes with a positive and dense surface thin film or nanofiltration membranes with a porous thin support.

## Introduction

Precise ion–ion separation has various applications including nutrient recovery from municipal wastewater, industrial wastewater reuse, and mineral resource extraction from brines.^1,2^ For example, lithium (Li) extraction from brine lakes is one of the most important separations to meet the growing demand in Li battery production for electric vehicles, mobile devices, and renewable energy storage systems.^3,4^ However, certain brine lake composition, in which the magnesium (Mg) to Li mass ratio (MLR) can be as high as 365:1, presents a significant challenge in the production of high-purity Li products.^4,5^ Therefore, the development of efficient and cost-effective technologies for the selective Li/Mg separation is important. Membrane-based processes such as electrodialysis (ED) and nanofiltration (NF) have emerged as promising alternatives to conventional Li extraction methods, such as solvent extraction and lime-soda precipitation, which are often inefficient and have significant environmental impacts.^6,7^ Although direct lithium extraction is challenging for ED and NF due to the high salinity and scaling potential, membrane-based separation techniques integrated with proper pre- and post-treatment units can achieve excellent selectivity when handling brines with a high MLR while minimizing the generation of waste streams and reducing the overall chemical and energy consumption of the process.^8,9^ Additionally, the excellent modularity of ED and NF makes them attractive for developing Li extraction systems of different scale. Thus, extensive research efforts have been devoted to advancing the core components of ED and NF, i.e., cation exchange membranes (CEMs) and NF membranes (NFMs), toward a very high Li/Mg selectivity.^10−15^

**Figure 1:**
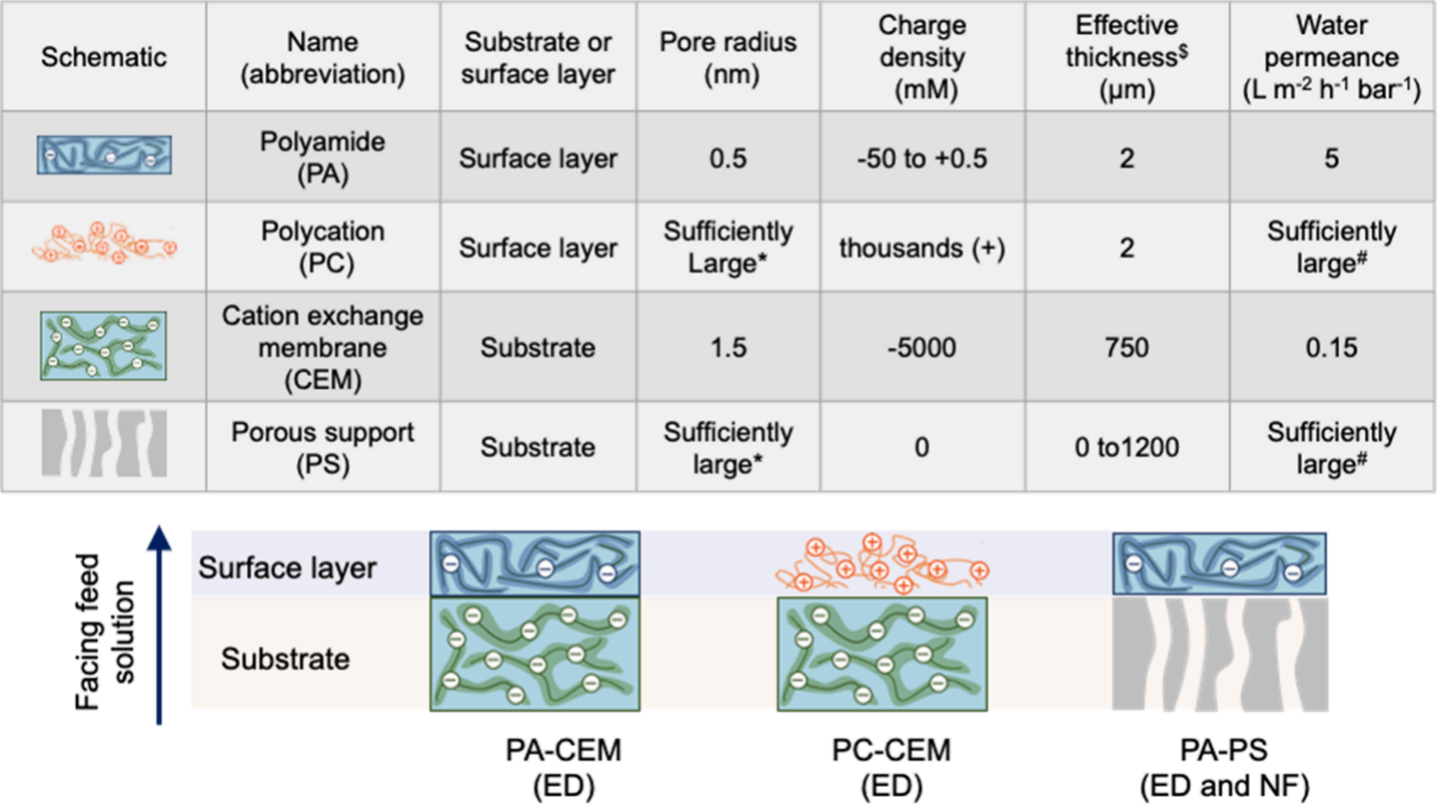
Theoretical constructs of three composite membranes investigated in this study. *: For pore radius, “sufficiently large” means that the non-Donnan partitioning effect and nonelectrostatic hindrance effect can both be ignored, i.e., ϕ_*i*_ = 1 in eq 7 and *K*_*i*_^ne^ = 1 in eq 8. ^$^: Effective thickness is defined as apparent thickness (*L*) divided by effective porosity (ε), which accounts for tortuosity and porosity. Herein, we assumed that *L* = 120 nm and ε = 0.06 for the PA film and *L* = 150 μm and ε = 0.2 for the CEM substrate. The PC film is assumed to have the same effective thickness as the PA film for fair comparison. ^#^: For water permeance, “sufficiently large” means that water transport resistance can be ignored compared to that of the other layer comprising the composite membrane.

Achieving excellent ion–ion separation in ED relies on ion transport across ion exchange membranes (IEMs) at different rates under the applied electric field, which in turn depends on the difference in ion–membrane affinity and ion mobility inside IEMs.^16^ Regular CEMs usually have little or no selectivity toward Li^+^ as the divalent counterion, Mg^2+^, is more preferably absorbed into the negatively charged membrane matrix, even though Li^+^ usually has a higher mobility than Mg^2+^. Thus, the development of monovalent selective CEMs is essential to enable ED for selective Li/Mg separation. Monovalent selective CEMs typically have an asymmetric composite structure, where the substrate is a regular CEM and the surface thin film can be a polymer layer with strong opposite charges (e.g., polyethylenimine, polyaniline, and quaternized chitosan) or a highly cross-linked dense layer, like a polyamide (PA) film (Figure 1).^16,17^ The key function of the surface thin film is to reduce the Mg^2+^ uptake into the substrate CEM via like-charge electrostatic repulsion or steric hindrance as Mg^2+^ carries more charges, has a larger hydrated radius (0.43 nm), and has a higher hydration energy (−1921 kJ mol^–1^), as compared to Li^+^ (0.38 nm and −519 kJ mol^–1^, respectively).^18^

Commercial monovalent selective CEMs (e.g., Selemion CSO from AGC, Japan, and Neosepta CIMS from ASTOM, Japan) with a surface thin film of abundant positive charges or polycations (PC-CEMs) have been widely studied and validated for their feasibility for separating Li^+^ from Mg^2+^.^9,19−24^ Lab-fabricated monovalent selective CEMs with a polyethylenimine surface thin film have shown a higher Li/Mg selectivity than commercial CEMs, likely due to a denser surface structure or a higher positive charge density.^25,26^ More recently, monovalent selective CEMs with a PA film (PA-CEM) have also been demonstrated with an improved monovalent/divalent selectivity.^27−29^ These different approaches to modify a CEM to improve its Li/Mg selectivity are summarized in Figure 1. We note that the monovalent selective surface layer is in contact with the feed solution when it is applied in the ED process.

Another class of membranes that has been explored in ED for selective Li extraction is NFMs that are traditionally used only in NF. Unlike CEMs, the support layers of the NFMs are porous and uncharged (or very weakly charged). The state-of-the-art NFMs are thin-film composite PA membranes made of an ultrathin PA film with subnanometer pores on top of a porous support (PA–PS in Figure 1, which will also be referred to as NFM interchangeably). The PA surface is weakly charged (or nearly uncharged) as compared to IEMs with a high fixed charge density. Divalent ions are usually better rejected than monovalent ions by NFMs in pressure-driven filtration because of (1) weaker partition of divalent ions to the PA matrix due to stronger steric, dielectric, dehydration, and Donnan effects of divalent ions and (2) more severe hindered transport of divalent ions inside the membrane pores.^30^ For the specific application of Li/Mg separation, positively charged NFMs, fabricated via coating positively charged polymers on the surface or introducing positively charged monomer to the interfacial polymerization step, have a higher Li/Mg selectivity than regular NFMs as a result of the stronger Donnan exclusion to Mg^2+^.^31−36^ NFMs have been proposed to replace commercial monovalent selective CEMs in the ED process to enhance the monovalent/divalent ion selectivity and reduce the membrane cost.^37−40^ Even though the uncharged support layer in NFMs also allows anions to permeate through, which may reduce charge efficiency, studies have also shown that using NFMs in the ED process does not require a higher energy consumption as compared to that using commercial monovalent selective CEMs.^37−40^

As composite monovalent selective CEMs with different types of surface thin films and NFMs have both been demonstrated to be effective for Li/Mg separation in selective ED processes, comparing the performance of these different membranes can guide the future design and optimization of Li-selective membranes for ED-based Li extraction. However, a fair comparison of different composite membranes can be challenging as the ion–ion separation performance depends on not only membrane structures and properties but also solution composition and operating conditions, which may vary substantially across different experimental studies.^41,42^ Although one can fabricate different types of membranes and perform systematic evaluation of their performance in Li/Mg separation using consistent conditions, the variability of membrane performance within the same membrane species due to the fabrication conditions can also introduce uncertainty to the performance comparison between different types of membranes. To address these experimental limitations, a theory-based systematic analysis can provide fundamental insights into designing membranes for ED-based Li/Mg separation.

NFMs and monovalent selective CEMs with different surface thin films can both be regarded as two-layer porous composite structures with each layer defined by layer thickness, effective pore size, and membrane charge density. Additionally, ion transport phenomena in IEMs and NFMs are fundamentally the same and can be described using the solution-friction model,^43−45^ which provides a unified framework to systematically compare the separation performance of different membranes via theoretical modeling to investigate the impact of membrane properties and operating conditions on membrane performance.

In this study, we first adapt the solution-friction model to describe ion transport across composite monovalent selective CEMs and NFMs in the ED processes under a unified framework. We then compare the Li/Mg separation performance of an ED process with monovalent selective CEMs with a surface thin film of strongly opposite charges or a PA film. We also demonstrate that the feed and receiving solution compositions have a strong impact on the separation performance. We further evaluate and compare the Li/Mg separation performance of PA-based NFMs in both ED and NF processes. Impacts of the surface thin film charge density of composite monovalent selective CEMs and NFMs are investigated at the coupon-scale with a detailed comparison of ion concentration profiles across the composite membranes and the flux contribution from each transport mechanism.

## Theory

### Solution-Friction Model for a Composite Membrane

The solution-friction model describes ion transport across the membranes starting with the Maxwell–Stefan theory, from which a force balance establishes between the chemical potential gradient of an ion species and the frictions between the ion species and all other components (i.e., ion–water, ion–membrane, and ion–ion frictions).^43−45^ Ion flux, *J*_*i*_ [mol m^–2^ s^–1^], after neglecting ion–ion frictions, can be equivalently described using the extended Nernst–Planck equation as proven in a previous study,^43^ which accounts for advection, diffusion, and electromigration mechanisms with hindrance:
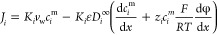
1where *v*_w_ [m s^–1^] is the water velocity across the membrane,  [m^2^ s^–1^] is the ion diffusion coefficient in an infinitely diluted solution,  [mol m^–3^] is the ion concentration of species *i* per volume of solution inside the membrane, *x* is the coordinate perpendicular to the membrane surface, ε is the effective porosity that accounts for porosity and tortuosity, *z*_*i*_ is the ion charge valence, and φ [V] is the electrical potential. *F* [96,487 C mol^–1^], *R* [8.314 J mol^–1^ K^–1^], and *T* [K] are the Faraday constant, ideal gas constant, and absolute temperature, respectively. *K*_*i*_ is the frictional or hindrance coefficient due to friction or interaction between species *i* and the membrane.

Ion partition at solution–membrane interfaces is assumed to be at local equilibrium. The partition coefficient, defined as the ratio of ion concentration near the interface inside the membrane () over that in the external bulk solution (), can be expressed as a product of the Donnan term that origins from membrane fixed charges and a non-Donnan term () that accounts for any other partition mechanism beside the Donnan effect:
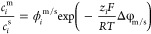
2where Δφ_m/s_ is the Donnan potential across the solution–membrane interface, and the solution phase can be either the feed or the receiving solution in ED. For a two-layer composite membrane, an additional interface exists between the substrate and the surface thin film. Similarly, the partition equilibrium can be expressed as
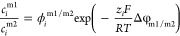
3where  and are interfacial ion concentrations in the surface film layer and the substrate layer, respectively. Δφ_m1/m2_ is the Donnan potential across the surface–substrate interface.  is the non-Donnan partition coefficient and can be expressed as the ratio of  and , i.e.,  = , imaging that a hypothetical external bulk solution phase is in equilibrium with both layers.^46^

Local charge neutrality is maintained at every position in each layer of the composite membrane:
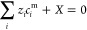
4where *X* [mol m^–3^] is the fixed charge density per volume of solution inside a layer. We note that *X* is assumed to be a constant and spatial variation is not considered in this study for simplicity, though a complex model can consider the possible inhomogeneous charge density distribution, and the divalent cation adsorption that may affect membrane charge density and even cause charge reversal.^47,48^ In addition, charge density is also affected by solution pH via the dissociation of functional groups in the polymer network,^49^ which we do not model specifically in this study, and thus any pH effect on the charge density is reflected by the variation of charge density itself directly.

For an ED process driven by an applied electric field, the current density (*I*_d_ [A m^–2^]) is contributed by fluxes of all mobile species:
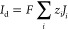
5

The solution-friction model describes water transport across the membrane by balancing the total pressure gradient with frictions between water–membrane and water–ions pairs:
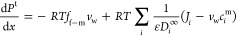
6where *P*^t^ [Pa] is the total pressure, defined as hydraulic pressure minus osmotic pressure. *f*_f–m_ [mol s m^–5^] is the friction coefficient between water and the membrane and can be related to the more commonly used pure water permeability, *A*_w_ [m s^–1^ Pa^–1^], by *f*_f–m_ = (*A*_w_*RTL*/ε)^−1^, where *L* is the membrane thickness. The solution-friction model has been applied to fit experimental data of ED, NF, and reverse osmosis with the non-Donnan partition coefficients and hindrance coefficients of ions as fitting parameters.^43−45,47^ However, explicit expressions relating these coefficients to ion and membrane properties with reasonable physical assumptions would be beneficial to theoretically investigating the ion–ion separation performance of membranes with different membrane properties.

### Non-Donnan Partition Coefficients

The non-Donan partition terms in eqs 2 and 3 have been interpreted with different theories for NFMs and IEMs. As NFMs have pore sizes close to the ion hydrated radius, the non-Donnan coefficients have been explained as a synergetic effect of steric and dielectric exclusions and also ion partial dehydration.^50−53^ Steric exclusion accounts for the fact that ions have finite volume and the probability for ions smaller than the membrane pore size to enter the pore is lower than 100%.^30^ The successful entry probability depends on the ratio of the ion radius over the effective pore radius. Dielectric exclusion imposes a solvation energy barrier to ions, which stems from the water dielectric constant reduction under nanoconfinement inside the subnanometer membrane pores and is thus a function of membrane pore size. Ion partial dehydration has recently been suggested to contribute to a substantial energy penalty when the hydrated ions shred off several water molecules during partition, but this effect has never been quantitatively described. IEMs have a much higher charge density and can also be regarded as a porous structure with larger effective pore radius (e.g., 1.0–3.0 nm) than NFMs (0.5–1.0 nm).^54−57^ The non-Donnan partition coefficients are often interpreted as ion–membrane affinity in the IEM transport theory, which accounts for any ion specific interaction with membrane functional groups beyond the Donnan effect.^44,58^ Manning’s counterion condensation theory is an alternative and more mechanistic interpretation.^59−61^ The non-Donnan term helps correct the overestimation of multivalent counterion uptake by the ideal Donnan model (i.e., only considers the Donnan equilibrium during ion partition).

To compare the nanoporous thin films and IEMs under a unified framework, we herein estimate the non-Donnan partition coefficients following the approach adopted in the Donnan steric pore model with dielectric exclusion (DSPM-DE)^30^:
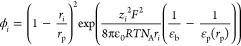
7where *r*_*i*_ [m] is the Stokes radius of ion *i,r*_p_ [m] is the effective membrane pore radius, ε_0_ is the dielectric constant of vacuum, *N*_A_ is Avogadro's constant. ε_b_ and ε_p_ are the relative dielectric constants of water in the bulk (ε_b_ = 78.4 for water at 25 °C) and in the pore, respectively, and ε_p_ is a function of *r*_p_. ϕ_*i*_ decreases sharply with pore radius when the pore radius is smaller than 1.0 nm, especially for divalent ions (Figure S1). We note that ion partial dehydration may have been accounted for to some extent in eq 7 as the dehydration mechanism overlaps with the steric and dielectric exclusions when involving ion size and solvation energy change. Moreover, the dielectric exclusion also mathematically helps avoiding the overestimation of multivalent counterion partition into IEMs as predicted by the pure Donnan effect.

### Hindrance Coefficients

Ion transport inside the membrane is hindered due to ion–membrane frictions, and the hindrance coefficients are often fitted from the experimental data. In the NF transport theory, like the DSPM-DE model, the ion–membrane friction is interpreted as physical collision of ions with the membrane pore wall, and the hindrance coefficients are estimated using expressions derived from the hydrodynamic theory (eq 8), with the assumption of a single spherical solute transporting across a perfect cylindrical micropore^62,63^
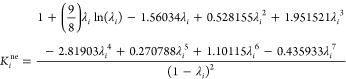
8where  is the nonelectrostatic hindrance coefficient and λ_*i*_ is the ratio of ion Stokes radius and membrane pore radius. We note that the validity of applying eq 8 to a subnanometer pore for charged ions remains questionable due to the simplifying assumptions of solute geometry and pore structure.^64^ In the IEM transport theory, ion diffusivity reduction inside the IEMs, especially for counterions, is also affected by a strong electrostatic effect besides the spatial effect (i.e., tortuosity and porosity). The electrostatic effect origins from the high charge density of IEMs and has been described by Manning’s counterion condensation theory.^59,65,66^ Fan et al. have proposed a simple expression to correlate the electrostatic hindrance coefficients of counterions, , to ion valence for a given IEM^65^:
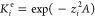
9where *A* may be fitted from experimental results and has been related to membrane charge density, *A* ≈ 0.003|*X*|^2/3^. Electrostatic hindrance to co-ion transport is neglected in this study (i.e., for co-ions).

To reconcile the NF and IEM transport theory, we herein estimate *K*_*i*_ in eq 1 as a product of  and , i.e., . We note that the combination treatment makes sense because (1) for weakly charged NFMs, the nonelectrostatic hindrance effect dominates *K*_*i*_ as  approaches unity (Figures S2 and S3); (2) for IEMs with a pore radius of 1–3 nm and a high charge density, electrostatic hindrance is much more significant (the “smaller”, the more significant, as “no effect” is defined by a factor of 1), especially for divalent ions (Figures S2 and S3).

### Modeling the Coupon-Scale Li/Mg Separation Performance

The adapted solution-friction model is applied to each layer of the composite membrane. Each layer of the membrane is characterized by a pore radius, a charge density, and an effective thickness (Figure 1). For the PA thin film, the selected structure property values (i.e., a pore radius of 0.5 nm, a charge density of −50 to 0.5 mM, and an effective thickness of 2.0 μm) are within the fitting results of different NFMs using the DSPM-DE model reported in a recent study.^67^ We note that the asymmetric value range of membrane charge densities is due to the fact that NFMs are usually negatively charged due to the abundance of carboxylic acid groups. The CEM layer is modeled as a thick, porous structure with a high density of negative charge. For the surface thin film with strong positive charges and the porous support membrane, we do not consider any non-Donnan partition effect and nonelectrostatic hindrance effect. Experimental characterization of the surface thin films on the composite CEMs is challenging because there is no convenient way of separating these surface layers from the substrate without altering their properties. Additionally, the ion exchange capacity of the surface film is negligible compared to the CEM substrate if the titration method is applied to measure the charge density of the composite membrane. Moreover, the conventional way of characterizing NFMs, such as using the rejections of neutral solutes to evaluate the molecular weight cutoff, cannot be applied to obtain the pore size of the surface PA layer of PA-CEMs, owing to the difficulty of filtering water through the composite CEMs with a very low water permeance.

Li/Mg separation using ED was modeled at the coupon scale (i.e., we only consider one-dimensional mass transfer in the direction perpendicular to the membrane surface). We modeled a feed solution that consists of 0.1 M LiCl and 0.1 M MgCl_2_ unless otherwise stated, representing the composition of elution solutions from a Li-selective adsorption pretreatment unit.^68^ The ED-based Li extraction process also requires a receiving stream that is different from the feed solution, which we assume to be 0.1 M NaCl, unless stated otherwise. We note that NaCl can be used as the receiving solution because Li^+^ will preferentially precipitate (as LiOH or Li_2_CO_3_) while Na^+^ will remain soluble in the subsequent chemical precipitation process. Additionally, an electrolyte solution is required as the receiving solution to reduce the solution resistance in ED. The impact of feed and receiving solution composition on separation performance is also discussed in this study, where ED-based Li/Mg separation using another feed (0.01 M LiCl and 0.1 M MgCl_2_) and two other receiving solutions (0.1 M LiCl and 0.01 M NaCl) were modeled.

For the coupon-scale ED performance analysis, the solution-friction model (eqs 1–6) was solved to determine the transmembrane ion flux as a function of applied current density (0–100 A m^–2^). Both external concentration polarization near solution-membrane interfaces (Text S1) and internal concentration polarization inside the porous support were considered. Coupon-scale Li/Mg separation performance was evaluated mainly based on two metrics: Li/Mg selectivity and Li flux. The Li/Mg selectivity or separation factor, *S*_Li/Mg_, is defined as bulk feed concentration ()-normalized ion flux ratio^41^
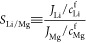
10where *J*_Li_ and *J*_Mg_ are the fluxes of Li^+^ and Mg^2+^ across the membranes.

## Results and Discussion

### Performance of Monovalent Selective CEMs in ED

**Figure 2:**
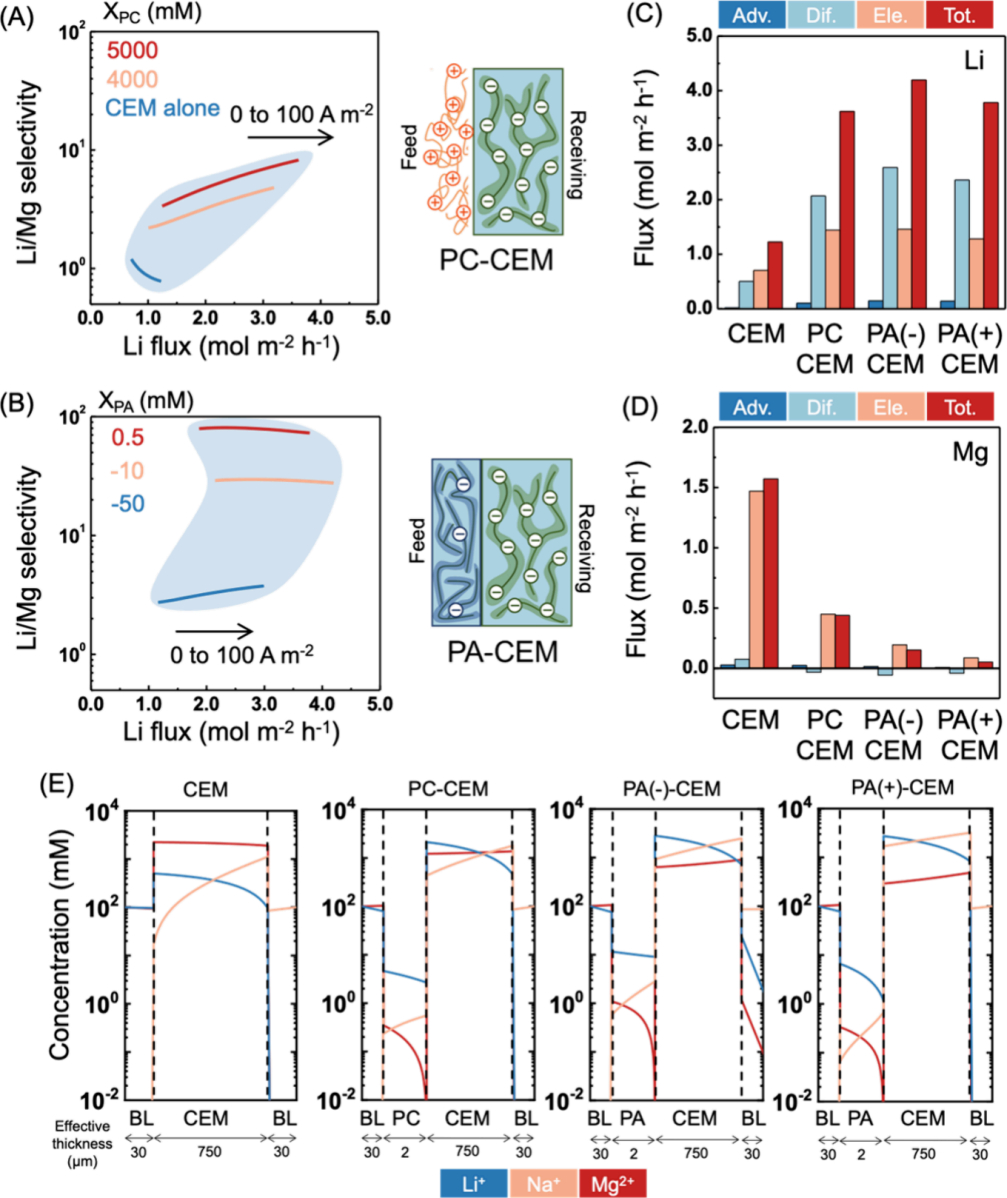
Performance of composite CEMs in ED. (A, B) Li/Mg selectivity versus Li flux as a function of current density and surface thin film (i.e., (A) positively charged thin film and (B) PA thin film) charge density. *X*_PC_ and *X*_PA_ are the charge densities of polycation (PC) and PA thin films, respectively. The current density increased from 0 to 100 A m^–2^ from left to right along each curve. The blue-gray area is just to guide the eyes, indicating the ranges of attainable selectivities and fluxes for each type of composite membrane when the surface film charge density and current density within the specified range are used for simulation. (C, D) Average Li and Mg flux contributions from advection (Adv.), diffusion (Dif.), electromigration (Ele.), and total flux (Tot.) in the CEM layer. (E) Concentration profiles of cations across the standalone CEM and composite CEMs. The *x*-axis is rescaled for a better presentation of each layer and does not reflect the actual layer thickness. “BL” stands for boundary layer. We note that the Li and Mg concentrations in the receiving solution are set to zero and are thus not shown in the receiving solution BL. For (C–E), the current density is 100 A m^–2^, the PC-CEM has a surface thin film with 5000 mM charge density, the PA(−)-CEM has a PA thin film with −50 mM charge, and the PA(+)-CEM has a PA thin film with 0.5 mM charge.

The coupon-scale Li/Mg separation performance of composite monovalent selective CEMs in the ED process was evaluated and compared. The composite monovalent selective CEMs that we discussed here include CEMs with either a relatively loose surface thin film of strong positive charges (PC-CEM) or a relatively dense but weakly charged PA thin film (PA-CEM). The regular CEM, as a reference, barely has selectivity to Li^+^ and the Li/Mg selectivity decreases to below one with an increasing current density (Figure 2A, blue curve). The loose surface thin film with positive charges enhances Li/Mg selectivity and increases Li flux under the same current density. Since the properties of the positively charged surface thin film are hard to characterize (as a separate layer from the substrate) and are thus rarely reported, we herein assumed no other exclusion or hindrance mechanisms in the surface thin film to highlight the electrostatic charge repulsion effect of positive charges. Therefore, the enhancement of selectivity and flux is not substantial until the positive charge density of the surface thin film is close to the negative charge density of the substrate CEM, with which the Li/Mg selectivity ranges from 3 to 10 (Figure 2A).

We note that the monovalent/divalent cation selectivity of commercial monovalent selective CEMs reported in the literature varies over a wide range from 1.2 to 50 as different solution compositions and operating conditions have been used.^19−24^ Coexistence of monovalent cations (e.g., K^+^ and Na^+^) tends to reduce the Li/Mg selectivity, while divalent ions, either cation or anion (e.g., Ca^2+^ and SO_4_^2–^), tend to increase the selectivity.^20,24^ Practically, the surface thin film may have a denser structure than what is assumed here and thus can have a larger Li/Mg selectivity due to the ion size sieving effect when the surface positive charge density is not as high as the substrate CEM.

PA-CEMs show several times to over an order of magnitude higher Li/Mg selectivity than PC-CEMs, depending on the charge density of the PA thin film (Figure 2B), because the denser PA surface thin film on the PA-CEM is more effective in reducing Mg^2+^ uptake and flux via steric and hydrodynamic hindrance than the loose positively charged polymer film on the PC-CEM. The presence of surface thin film increases Li^+^ flux while reduces Mg^2+^ flux as compared to the standalone CEM (Figure 2C,D). A weakly positively charged PA film has a slightly lower Li^+^ flux than that of a weakly negatively charged PA film under the same current density, but it rejects Mg^2+^ more effectively. By decomposing the average ion flux across the CEM layer to different transport mechanisms, our analysis reveals that Li^+^ flux is contributed mainly by electromigration and diffusion, while Mg^2+^ flux is dominated by electromigration, which is proportional to the Mg^2+^ concentration inside the CEM layer. Therefore, the reduction of the Mg^2+^ flux in a composite CEM is mainly due to the reduction of Mg^2+^ uptake to the CEM by the presence of the surface thin film. This argument can be verified with ion concentration profiles across the composite membranes (Figure 2E). Although the MLR is ∼3.4 in the bulk feed solution, partition of Mg^2+^ into the surface thin film is unfavorable due to like-charge electrostatic repulsion and/or steric and dielectric exclusions, which results in a Mg^2+^ concentration that is much lower than Li^+^ in the surface thin film. Moreover, Mg^2+^ depletion occurs near the interface between the surface thin film and the substrate layer, which further reduces the Mg^2+^ uptake into the substrate CEM. For example, the Mg^2+^ concentration in the CEM layer of the PA(+)-CEM with positively charged PA is over an order of magnitude lower than that in the standalone CEM. Meanwhile, more Li^+^ and Na^+^ partition into the CEM layer to maintain charge neutrality with enhanced Li^+^ and Na^+^ flux to carry the applied current. We further found that a thicker surface thin film (either the loose positively charged film or the denser PA film) and a PA film with a smaller pore size will increase the Li/Mg selectivity (Figures S4 and S5) because Mg^2+^ encounters more transport resistance in both scenarios.

**Figure 3:**
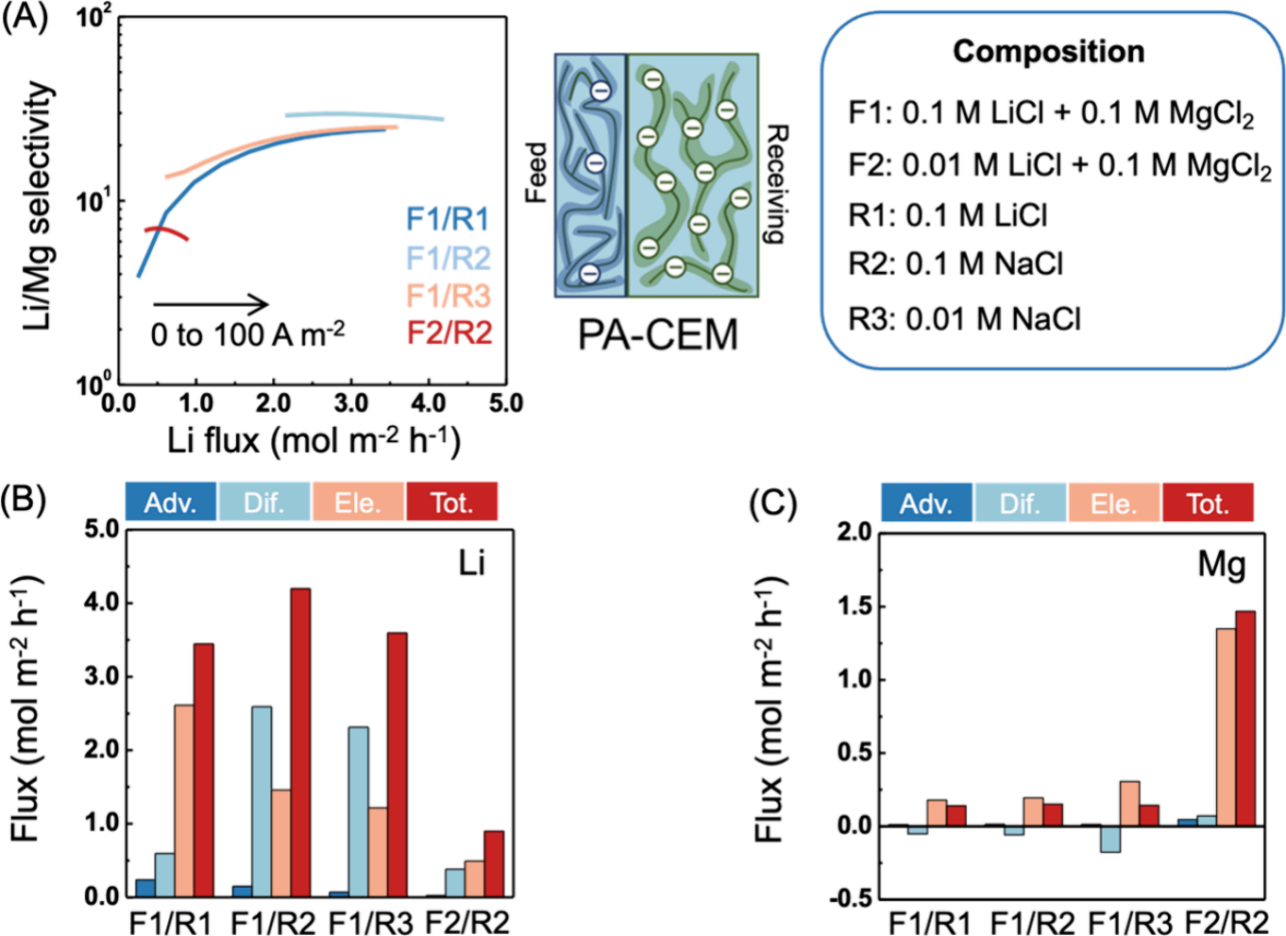
Impact of feed and receiving solution composition on the performance of PA-coated CEM. (A) Li/Mg selectivity vs Li flux as a function of current density and solution composition. The current density increased from 0 to 100 A m^–2^ from left to right along each curve. (B, C) Average Li and Mg flux contributions from advection (Adv.), diffusion (Dif.), electromigration (Ele.), and total flux (Tot.) in the CEM layer. For (B) and (C), the current density is 100 A m^–2^, and PA-CEM has a PA thin film with −10 mM charge density.

Solution composition and salinity can substantially affect the performance of IEMs, like perm-selectivity, conductivity, and ion–ion selectivity.^42,69−71^ Specifically, the compositions of the feed and receiving solutions affect the ED-based Li/Mg separation performance because (1) the ion diffusion flux is driven by the concentration gradient and (2) ion electromigration flux is proportional to the ion concentration inside the membrane, which is in turn proportional to the bulk concentrations via partition. Using PA-CEM as an example, we evaluated and compared the separation performance under two feed solutions and three receiving solutions (Figure 3A). The corresponding ion concentration distributions of each scenario can be found in the Supporting Information (Figure S6). When the feed contains 0.1 M LiCl and 0.1 M MgCl_2_ (i.e., F1), a Li-free receiving solution (e.g., R2 and R3) benefits the Li^+^ diffusive flux across the membrane and thus results in both higher Li^+^ flux and Li/Mg selectivity than using a receiving solution that contains the same Li^+^ concentration as the feed (i.e., R1) (Figure 3B).

When using NaCl as the receiving solution, a higher NaCl concentration leads to both higher Li^+^ flux and Li/Mg selectivity especially when the current density is low (Figure 3A). This is because the Na^+^ diffusive flux is in the opposite direction to the Li^+^ flux, which facilitates the exchange of Li^+^ from the feed to the receiving solution to maintain the Donnan equilibrium (i.e., the working principle of diffusion dialysis). As both diffusion flux and electromigration flux depend strongly on the feed concentration, when changing to a feed solution containing much less Li^+^ (e.g., F2), Li^+^ flux is reduced substantially while Mg^2+^ flux increases to maintain the same current density (Figure 3C), which also results in a much lower Li/Mg selectivity. Therefore, it is unfair and meaningless to compare the separation performance of different membranes in the literature evaluated using different solutions and operating conditions.

### Performance of NFMs in ED

**Figure 4:**
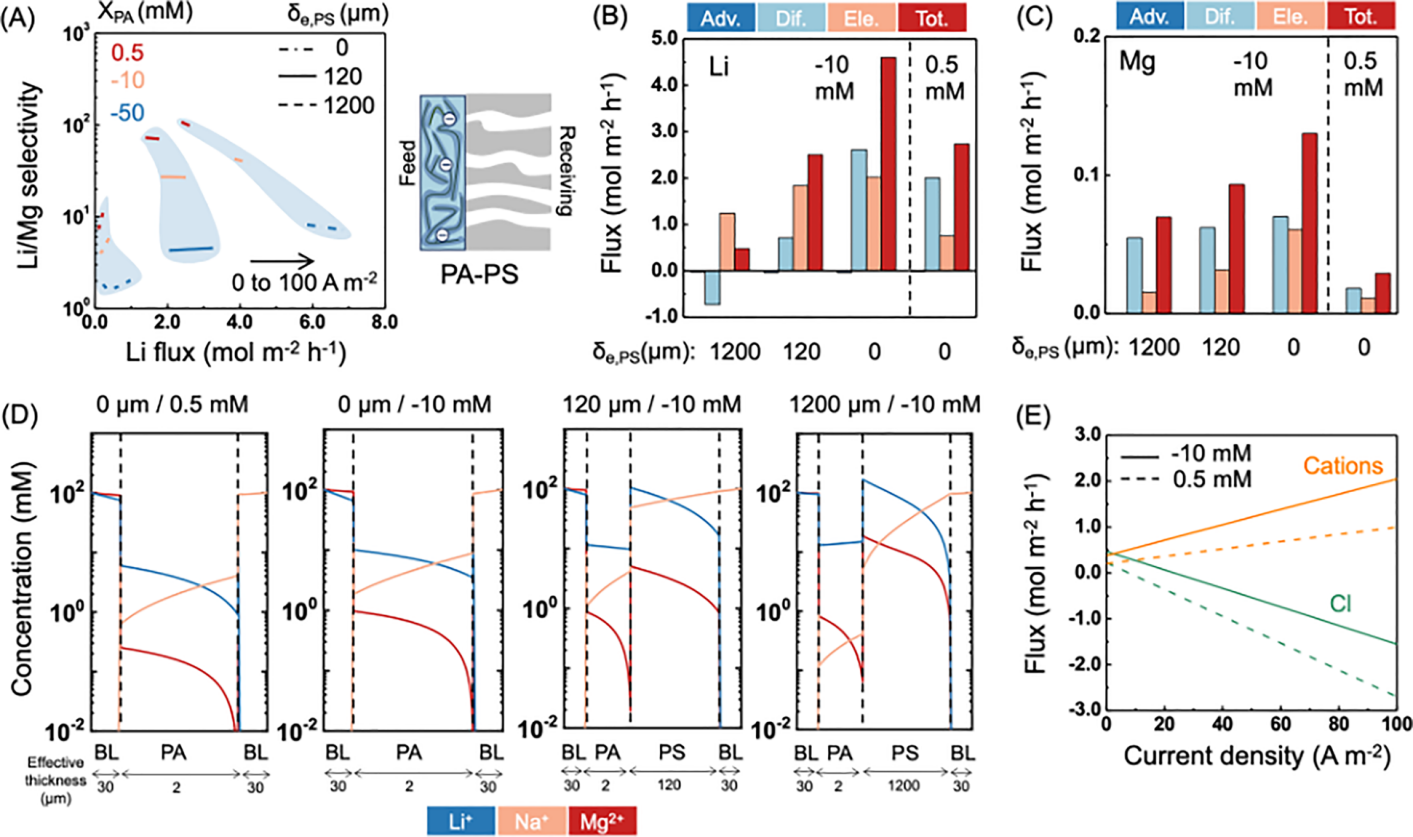
Performance of NFMs in ED. (A) Li/Mg selectivity versus Li flux in ED as a function of current density, PA thin film charge density, and support layer effective thickness. *X*_PA_ is the charge density of PA thin films. δ_e,PS_ is the effective thickness of the porous support membrane. The current density increased from 0 to 100 A m^–2^ from left to right along each curve. The blue-gray area is just to guide the eyes, indicating the ranges of attainable selectivities and fluxes for NFMs when the surface PA film charge density and current density within the specified range are used for simulation. (B, C) Average Li and Mg flux contributions from advection (Adv.), diffusion (Dif.), electromigration (Ele.) and total flux (Tot.) in the PA thin film of the NFMs. (D) Concentration profiles of cations inside the NFMs. The *x*-axis is rescaled for a better presentation of each layer and does not reflect the actual layer thickness. “BL” stands for boundary layer. For (B–D), the current density in ED is 100 A m^–2^. (E) Cation and chloride flux as a function of current density when across a standalone PA thin film.

Li/Mg selectivity of the composite NF membrane in the ED process varies by about 2 orders of magnitude from ∼2 to ∼100, depending on the PA film charge density and the effective thickness of the porous support membrane (Figure 4A). Both Li/Mg selectivity and Li^+^ flux decrease with increasing effective thickness of the porous support due to enhanced internal concentration polarization. For a support-free PA thin film, the Li^+^ diffusion flux is even larger than its electromigration flux under the given bulk solution compositions and current density (Figure 4B). But, the Li^+^ diffusion flux decreases substantially in the presence of a support layer as in real NFMs.

As the support layer becomes thicker (from left to right in Figure 4D), the Li^+^ concentration gradient in the PA film gradually diminishes (see blue curves in the PA layers in Figure 4D) and even reverses in direction when the support layer is 1200 μm, which leads to a negative diffusion flux with a support layer that is 1200 μm thick (Figure 4B). Although a thinner support layer also promotes more unfavorable permeation of Mg^2+^ (Figure 4C), the support-free membrane still shows the highest Li/Mg selectivity due to the higher permeability of Li^+^.

On the one hand, an ultrathin (20–200 nm) standalone PA film is too fragile to be used in any practical context despite the excellent selectivity (e.g., ∼100) and high Li^+^ flux achieved by a support-free PA thin film. On the other hand, commercial NFMs usually have a thick, tortuous, and low-porosity polysulfone or poly(ether sulfone) support layer that often sits on another nonwoven fabric. Such a support structure results in an effective thickness of thousands of micrometers, which hinders ion transport and, in turn, substantially compromises both Li/Mg selectivity and Li^+^ flux. Thus, a thin support layer with a high porosity and a low tortuosity (e.g., with ∼120 μm effective thickness) is necessary for NFMs to be an effective alternative to monovalent selective CEMs in the ED-based Li/Mg separation process. Support layers with such structural properties have been developed for forward osmosis to mitigate internal concentration polarization^72^ and can also be used to develop NFMs for ED-based Li/Mg separation. The primary difference is that here, we need a PA thin film that is permeable to monovalent ions but rejects divalent ions instead of one that universally rejects all ions as in forward osmosis membranes.

When it comes to the effect of surface charge of PA NFMs on Li/Mg separation, a less negative or slightly positive PA thin film substantially increases Li/Mg selectivity under the same current density but at the cost of substantial Li^+^ flux reduction (which also means a reduction of current efficiency), especially for the support-free membrane (Figure 4A). This is a result of an enhanced Cl^–^ flux in the opposite direction (i.e., from receiving solution to feed solution) across the NFM when the PA film becomes positively charged (Figure 4E). Here, a major difference between the NFM and composite CEM is that the CEM excludes co-ions, whereas the support layer of the NFM has no ion selectivity. With composite monovalent selective CEMs, Cl^–^ is the co-ion to the substrate CEM and is thus strongly excluded and has little contribution to current. Therefore, the Li^+^ flux of a PA-CEM varies to a much lower degree when the charge density of PA film changes (Figure 2B). We further found that Li/Mg selectivity can be increased by having a thicker PA surface film and/or with smaller pore sizes (Figure S7), the same as the PC-CEMs and PA-CEMs.

### Performance of Composite Membranes in ED vs NF

**Figure 5:**
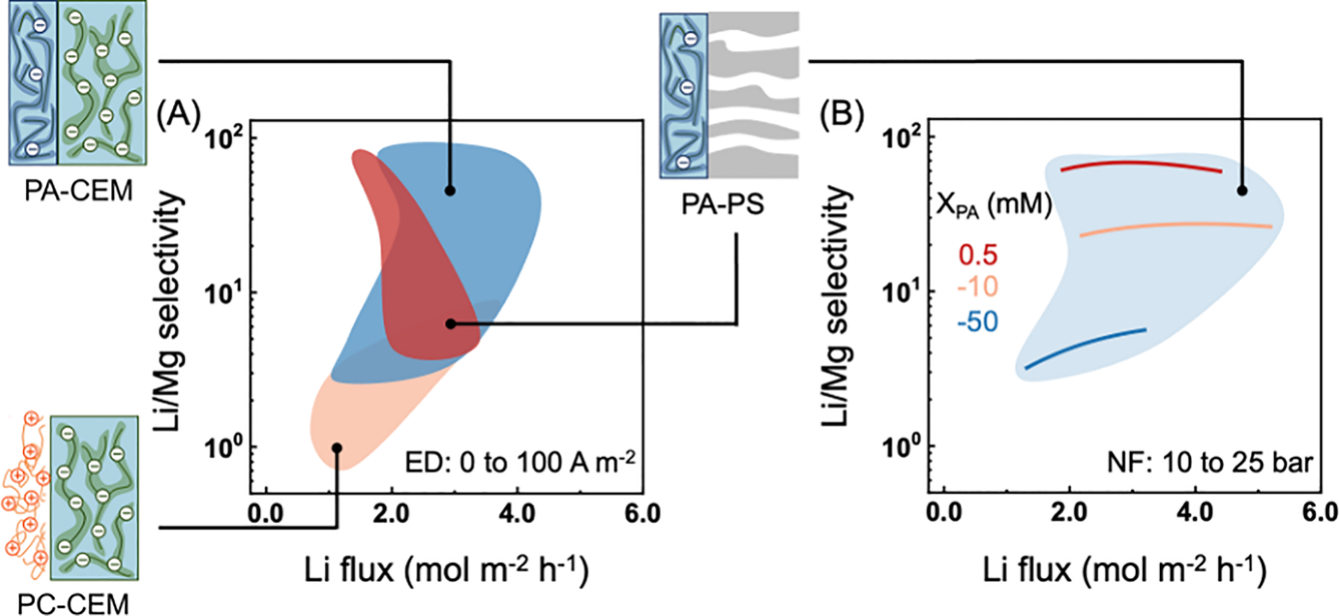
Coupon-scale performance comparison of composite membranes in ED versus in NF. (A) Li/Mg selectivity versus Li flux of PC-CEMs, PA-CEMs, and PA-PS (i.e., NFM) in ED. The current density varies from 0 to 100 A m^–2^. The PA thin film charge density varies from −50 to 0.5 mM. The positively charged thin film charge density varies from 0 to 5000 mM. NFMs have a porous support with an effective thickness of 120 μm. (B) Li/Mg selectivity versus Li flux of NFMs in the NF as a function of pressure and PA thin film charge density. The blue-gray area is just to guide the eyes, indicating the ranges of attainable selectivities and fluxes for NFMs when the surface PA film charge density and pressure within the specified range are used for simulation.

The coupon-scale performance evaluation indicates that a positively charged surface thin film is always beneficial to Li/Mg separation regardless of the substrate membrane (e.g., the CEM or uncharged porous support). PA-based NFMs (i.e., PA-PS) and PA-CEMs have a higher Li/Mg selectivity than PC-CEMs (Figure 5A), indicating that ion size-related effects, e.g., steric and dielectric exclusions induced by nanoconfinement, are more effective than like-charge electrostatic repulsion for enhancing Li/Mg selectivity. The performance of replacing monovalent CEMs with NFMs in the ED process strongly depends on the porous support structure. With practically achievable thin supports (as those that have been developed for forward osmosis), PA-based NFMs have a similar Li/Mg selectivity to PA-CEMs when used in ED (Figure 5A), though the Li^+^ current efficiency (or flux) is limited due to the unavoidable counterion (e.g., Cl^–^) flux in the reverse direction as discussed previously.

To answer the question whether NFMs have any performance advantage when used in NF (i.e., pressure-driven filtration) over NFMs and PA-CEMs in the ED process, we further evaluated the separation performance of NFMs in NF. We found that PA-based NFMs when used in the NF process show a Li/Mg selectivity similar to that in the ED process, though the driving forces are fundamentally different (Figure 5B). When used in NF, the Li/Mg selectivity of NFMs is theoretically independent of the support layer thickness as there is no concentration polarization on the permeate side either externally in the bulk or internally in the porous support.

Li/Mg selectivity is sensitive to Mg^2+^ rejection. The Mg^2+^ rejection is over 98% when the PA has a charge density of 0.5 mM and is lower than 92% when the charge density is −50 mM. The Li^+^ rejection of the positively charged membrane is lower than that of a negatively charged membrane (Figure S8), mainly due to the fact that more Li^+^ ions need to permeate through the membrane to maintain charge neutrality when Mg^2+^ ions are strongly rejected.^73^ A high pressure or permeate flux may lead to selectivity reduction due to an enhanced external concentration polarization in the feed.^74^ When the PA thin film is negatively charged and Li^+^ is the counterion, Li flux is dominated by electromigration; with a positively charged PA thin film where Li^+^ is the co-ion, Li flux is dominated by diffusion (Figure S9). Mg^2+^ flux is controlled by both diffusion and electromigration, but its transport is substantially slower than that of Li^+^ due to steric hindrance and is hindered to an even greater extent when the PA thin film is positively charged (Figure S9).

We note that the performance comparison of different composite membranes is limited only to the coupon scale. Module-scale performance can be very different as the feed solution composition varies along the module in a continuous single-pass ED or NF process. For example, the MLR of the feed solution increases along the module in both processes but for different reasons. In ED, the increase in MLR is caused by the depletion of Li^+^ ions as they are selectively transported to the receiving solution. Meanwhile, in NF, it is mainly due to the concentration of Mg^2+^ as water recovery increases. Moreover, Li recovery in the coupon-scale analysis is zero, while achieving high Li recovery is as important as high selectivity for a Li extraction process. Our previous analysis has shown that an operational trade-off between Li/Mg selectivity and Li recovery exists at the module-scale NF process, i.e., selectivity decreases as Li recovery increases.^74,75^ Therefore, it is worth extending the performance evaluation and comparison to the module scale in future study to see if the trade-off between Li/Mg selectivity and Li recovery still holds in ED-based separations.

## Implications

Selective ED is a promising membrane-based process to separate Li^+^ from Mg^2+^, which is the most critical step for Li extraction from brine lakes. In this study, we theoretically compared ED-based Li/Mg separations using different monovalent selective CEMs and NFMs with a unified solution-friction model. Our analysis has revealed several insights, which are summarized below:

- Monovalent selective CEMs with a dense surface thin film like PA is more effective in enhancing Li/Mg selectivity than those with a loose but highly charged thin film.
- PA film-coated CEMs when used in ED have a similar performance to PA-based NFMs when used in NF.
- For NFMs to replace monovalent selective CEMs in ED for Li/Mg separation, it is critical to use a thin support layer with low tortuosity and high porosity to reduce the internal concentration polarization.

Moreover, literature studies exploring the application of NFMs in selective ED usually prepare the PA films via a conventional interfacial polymerization technique with piperazine and trimesoyl chloride, which usually results in a negatively charged surface with a wide pore size distribution, unfavorable to the precise Li/Mg separation. We expect that PA thin films with more positive charges (e.g., by changing the monomer type or having a surface coating) and a more uniform pore size would improve the Li/Mg separation performance in both ED and NF. To our knowledge, no study has evaluated the Li/Mg separation performance of PA film-coated CEMs, which based on our analysis, are promising to achieve high selectivity and high Li flux simultaneously. However, it is unclear whether such PA-CEMs can readily be made following the protocols for fabricating PA-based NFMs reported in the literature since the properties of the support layer (e.g., CEM vs polysulfone support) may substantially change the interfacial polymerization process and thus the final PA layer properties. We also note that membranes with ion-specific channels or other separation mechanisms may achieve better separation performance than the composite membranes considered here and is thus worthy of future investigation and comparison.^14,76,77^ Furthermore, energy consumption and cost analysis are also necessary in future analysis for a comprehensive performance comparison, especially considering that one major benefit to substitute monovalent selective CEMs by NFMs is to reduce the membrane cost.

## Supporting Information Available

Concentration polarization (Text S1); non-Donnan partition coefficients as a function of pore radius (Figure S1); nonelectrostatic hindrance coefficients as a function of pore radius (Figure S2); electrostatic diffusion hindrance factor as a function of membrane charge density (Figure S3); effect of surface film thickness on the performance of PC-CEMs (Figure S4); effect of surface film thickness and pore size on the performance of PA-CEMs (Figure S5); concentration profiles in PA-CEMs under different external solution compositions (Figure S6); effect of surface film thickness and pore size on the performance of NFMs in ED (Figure S7); Li and Mg rejection in NF as a function of applied pressure (Figure S8); average Li and Mg flux contributions from different mechanisms in the PA thin film of the composite NFMs (Figure S9) (PDF)
