# Supplementary material for: Membrane Design Principles for Ion-Selective Electrodialysis: An Analysis for Li/Mg Separation
Source: Environ Sci Technol. 2024 Feb 7;58(7):3552–63. doi: 10.1021/acs.est.3c08956 (PMC10882969; doi:10.1021/acs.est.3c08956)
Supplement: Supplementary file 1 — es3c08956_si_001.pdf [file es3c08956_si_001.pdf]

Supporting Information for

Membrane Design Principles for Ion-selective Electrodialysis: Analysis for Li/Mg Separation

Environmental Science & Technology

Ruoyu Wang[a] and Shihong Lin* [a,b]

[a] Department of Civil and Environmental Engineering, Vanderbilt University, Nashville, Tennessee 37235-1831, USA

[b] Department of Chemical and Bimolecular Engineering, Vanderbilt University, Nashville, Tennessee 37235-1831, USA

*Email: shihong.lin@vanderbilt.edu

6 pages

1 Text

9 Figures

Text S1. Concentration polarization

The extended Nernst-Planck equation was solved in the boundary layer near the solution-membrane interface to account for external concentration polarization,

$$J_i = v_\mathrm{w} c_i^\mathrm{s,m} - \frac{D_i^\infty}{\delta_\mathrm{b}}\left(c_i^\mathrm{s,m} - c_i^\mathrm{s,b}\right) - z_i c_i^\mathrm{s,m} \frac{F}{RT}\xi \tag{S1}$$

where $c_i^\mathrm{s,m}$ and $c_i^\mathrm{s,b}$ are ion concentrations in the solution phase at the membrane interface and in the bulk, respectively. ξ is the electrical potential gradient at the interface. δ_b is the boundary layer thickness, which depends on flow hydrodynamics and may be estimated using Sherwood correlations. We assumed δ_b to be 30 μm in this study. Charge neutrality still applies at the solution-membrane interface.

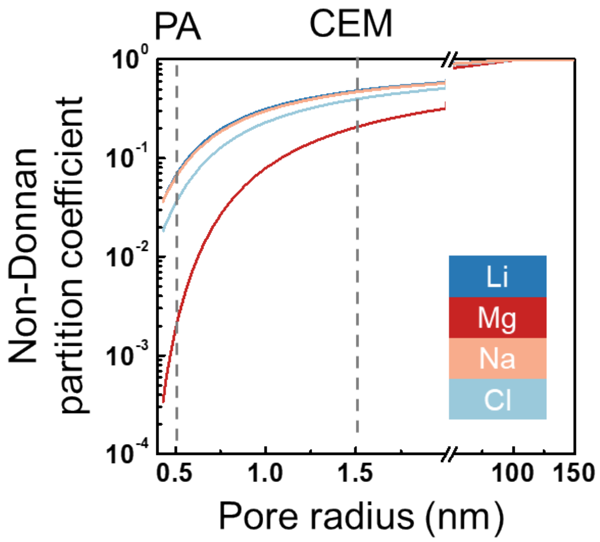

Fig.S1 Non-Donnan partition coefficients as a function of pore radius.

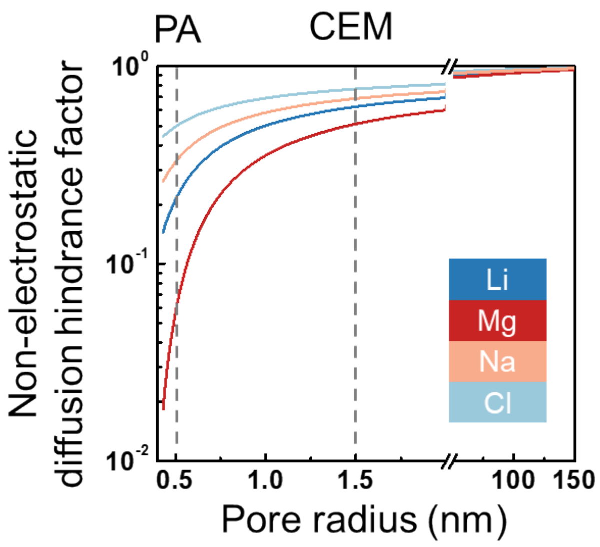

Fig.S2 Non-electrostatic hindrance coefficients as a function of pore radius.

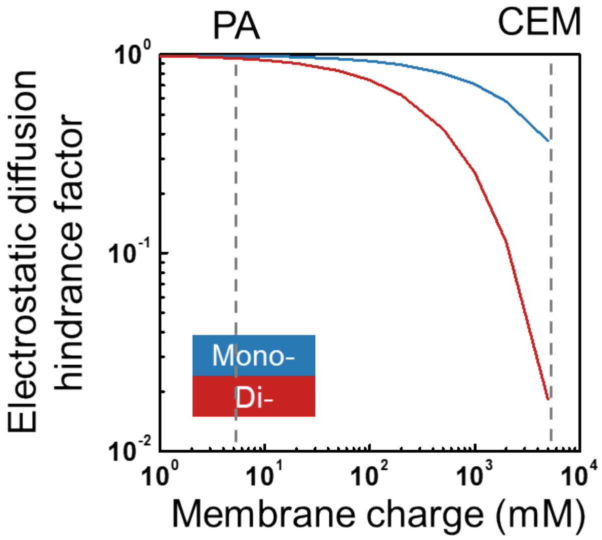

Fig.S3 Electrostatic diffusion hindrance factor as a function of membrane charge density.

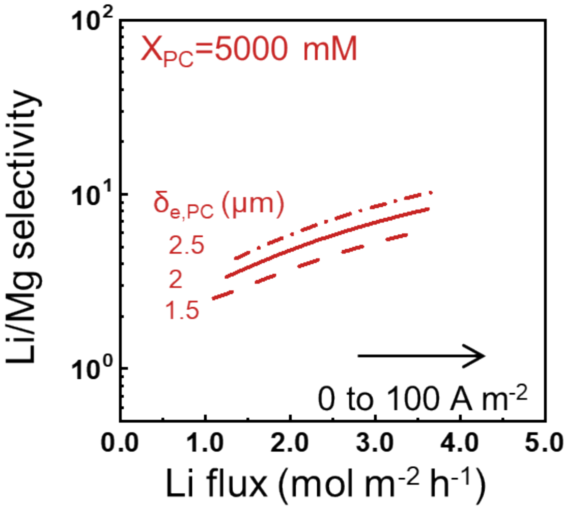

Fig.S4 Li/Mg selectivity versus Li flux in ED of PC-CEM as a function of current density at different surface layer thickness. $\delta_{e,\,PC}$ is the effective thickness of the surface thin film. The current density increased from 0 to 100 A m^{-2} from left to right along each curve.

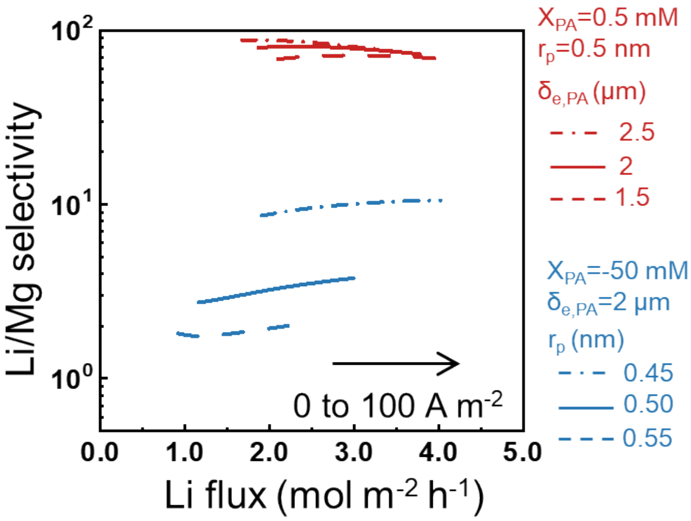

Fig.S5 Li/Mg selectivity versus Li flux in ED of PA-CEM as a function of current density at different surface layer thickness and pore radius. $\delta_{e,\,PA}$ and r_p is the effective thickness and pore radius of the surface PA thin film, respectively. The current density increased from 0 to 100 A m^{-2} from left to right along each curve.

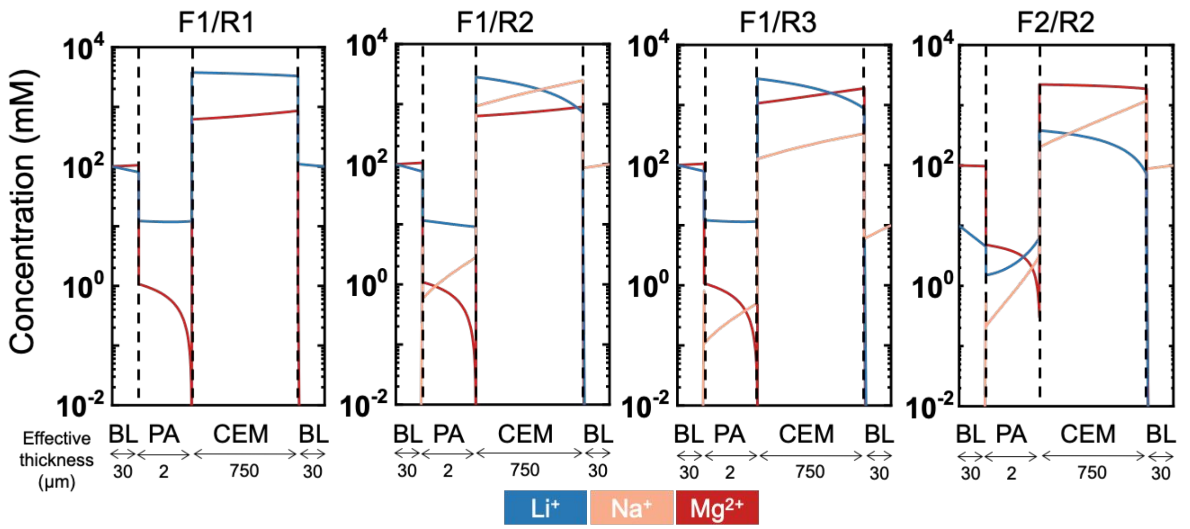

Fig.S6 Concentration profiles of cations across the composite CEMs. The x-axis is re-scaled for a better presentation of each layer and does NOT reflect the actual layer thickness. 'BL' stands for boundary layer.

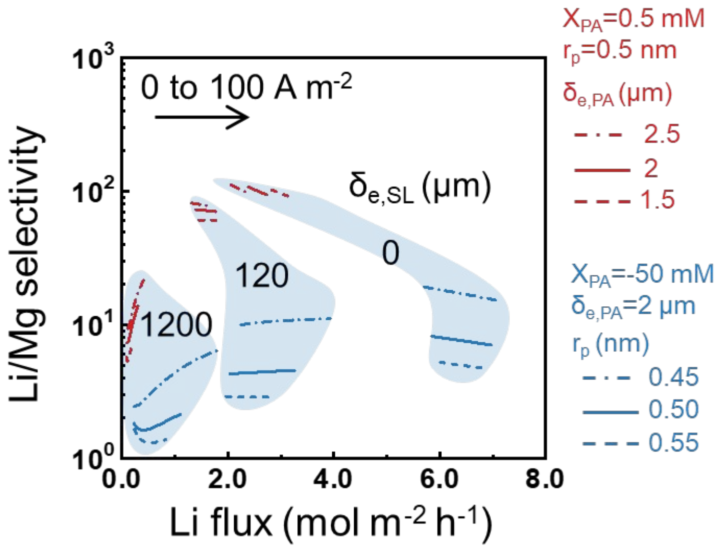

Fig.S7 Li/Mg selectivity versus Li flux in ED of PA based NFM as a function of current at different density support layer effective thickness, surface film effective thickness and surface film pore radius. $\delta_{e,\ PA}$ is the effective thickness of the porous support. $\delta_{e,\ PA}$ and r_p is the effective thickness and pore radius of the surface PA thin film, respectively. The current density increased from 0 to 100 A m^{-2} from left to right along each curve.

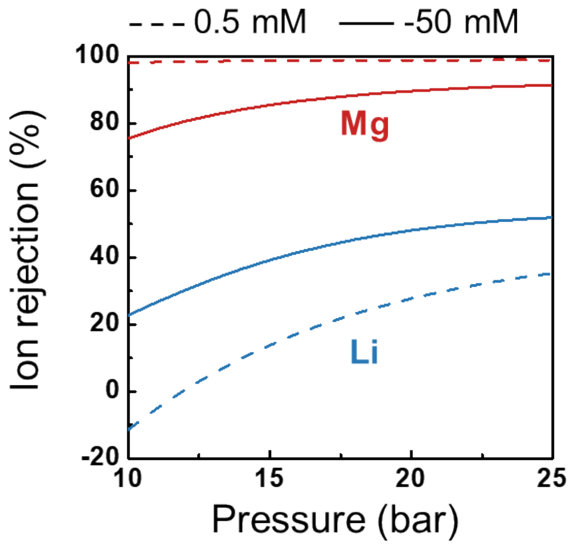

Fig.S8 Li and Mg rejection in NF as a function of applied pressure. Feed solution is 0.1 M LiCl and 0.1 M $MgCl_2$.

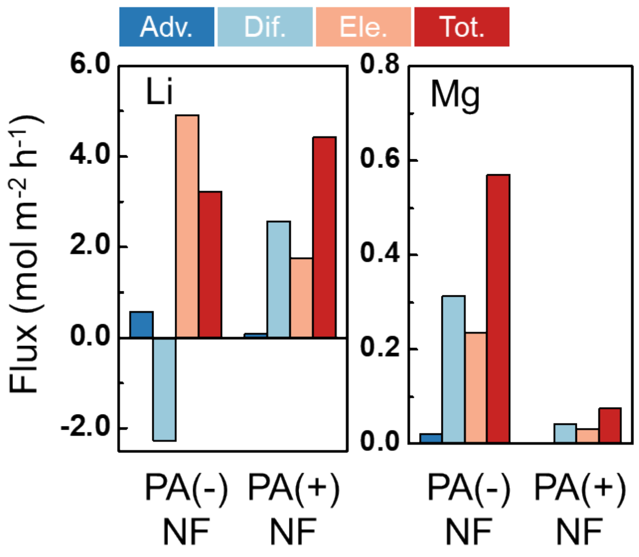

Fig.S9 Average Li and Mg flux contributions from advection, diffusion and electromigration in the PA thin film of the composite NFMs. Pressure is 25 bar. PA(-)NF and PA(+)NF represent PA films with -50 mM and 0.5 mM charge density, respectively.
